# Psychosocial stress and longitudinally measured gestational weight gain throughout pregnancy: The Ulm SPATZ Health Study

**DOI:** 10.1038/s41598-020-58808-8

**Published:** 2020-02-06

**Authors:** S. Braig, C. A. Logan, F. Reister, D. Rothenbacher, J. Genuneit

**Affiliations:** 1 Institute of Epidemiology and Medical Biometry, Ulm University, Ulm, Germany; 2 Department of Gynecology and Obstetrics, University Medical Center Ulm, Ulm, Germany; 3 Pediatric Epidemiology, Department of Pediatrics, Leipzig University Medicine, Leipzig, Germany

**Keywords:** Predictive markers, Risk factors

## Abstract

Psychosocial stress is thought to influence gestational weight gain (GWG), but results are inconsistent. We investigated the relationship of questionnaire-based maternal stress and related constructs assessed at childbirth with maternal weight measured throughout pregnancy. Data were derived from the Ulm SPATZ Health Study, a birth cohort recruited from the general population (04/2012–05/2013, Ulm, Germany). Adjusted generalized estimating equations were performed. Regression coefficients (b) and 95% confidence intervals, each highest versus lowest tertile of stress or related constructs, are presented. In 748 women, we observed positive associations for maternal chronic stress (b = 4.36 kg (1.77; 6.95)), depressive symptoms (b = 2.50 kg (0.14; 4.86)), anxiety symptoms (b = 3.26 kg (0.62, 5.89)), and hair cortisol (b = 3.35 kg (0.86; 5.83)) with maternal weight at the first gestational month. GWG was considerably lower in mothers with higher chronic stress. Pregnancy-related anxiety was positively related to weight at first month (b = 4.16 kg (1.74; 6.58)) and overall GWG. In contrast, no association was observed between anxiety symptoms and GWG. Odds ratios for association with inadequate weight gain according to Institute of Medicine recommended cutoffs differed from the results presented obove. There is evidence of an association between stress and weight gain lying beyond the recommended cut-offs, which however needs further corroboration.

## Introduction

Conditions during pregnancy play a major role in shaping not only aspects of fetal development and birth outcomes but also subsequent newborn, child, and adult health outcomes^1–4^. Maternal psychosocial stress has been frequently studied for its potential association with dietary behavior and/or changes in body weight, however results for these associations during pregnancy have been inconsistent.

There are several common conceptualizations of ‘psychosocial stress’ (from now on referred to as stress) which include: the occurrence of stressful situations or events, individual’s perception of stress following such circumstances, and the subsequent psychological consequences (e.g., depression, anxiety symptoms)^5^. One proposed mechanism linking maternal stress during pregnancy to maternal or child outcomes involves steroid hormones secreted by the hypothalamic‐pituitary‐adrenal (HPA) axis following stressful events. Prolonged activation of the HPA axis may promote abdominal visceral fat accumulation as well as an increase of body weight via increased cortisol secretion (e.g.,^6^).

Additionally, stress and related constructs appear to alter overall energy intake through under- or overeating, depending on the nature of the stressor, its severity as well as an individual predisposition^7^. In a cross-sectional study in pregnant women, emotional problems and negative attitudes towards the baby were related to dietary behavior, as was the pre-pregnancy body mass index^8^. Apart from stress, a meta-analysis suggests that depression is linked to weight and weight gain in a bidirectional manner^9^.

Nevertheless, evidence for an association between stress and related constructs and weight gain during pregnancy is mixed which may be due to different measurements of stress. One systematic review^10^ concluded that maternal depression, but not anxiety or stress during pregnancy appears to have a direct relationship with excessive gestational weight gain (GWG). Another systematic review^11^ reported that only one out of the seven included studies observed a negative association between depression and excessive GWG, whereas no link between stress or anxiety and excessive GWG was reported. Furthermore, in a large study of 13,314 mothers^11^, depression was not meaningfully associated with inadequate or excessive GWG, yet a later study^12^ reported a relationship beween anxiety and GWG in multiparous women only. Notably, most of the evidence is based on self-reported pre- and post-pregnancy body weight as well as self-reported body height with higher potential for misclassification than prospective measurement of maternal weight^13,14^.

Given previous inconsistent results, this study aims to investigate the association of stress, anxiety, and depression symptoms with GWG based on paper documentation of routine preventive medical examination including several measurements of body weight. We hypothesized that higher stressed mothers would gain more or less weight than lower stressed mothers due to mechanisms e.g., like a change in the HPA axis or changed dietary behaviors. Thereby, hair cortisol concentrations (HCC) were measured as a potential biomarker of HPA axis activation. The assessment of the biological stress response by using HCC has gained interest, as it offers a way to assess aggregated, long-term cortisol levels with a single non-invasive sampling^15–17^. By capitalizing on the continuous incorporation of lipophilic substances into the slowly growing hair matrix, HCC are assumed to provide an easily obtainable index of hormone levels integrated over the periods of several months. HCC may thus be more representative of normal, long-term cortisol secretion than single measures in saliva, blood, or urine. We further analyzed the associations of self-reported stress or related constructs with GWG above or below cut-offs recommended by the Institute of Medicine (IOM)^18^ to compare the results.

## Results

Following restriction to singleton full-term deliveries without maternal diabetes mellitus diagnosis or gestational diabetes (n = 107 excluded) or last obstetrician examination before gestational day 245 (n = 57 excluded), information was available for 748 mothers of whom the majority (70.3%) were between 26 and 35 years of age and of German nationality (85.4%) (see Table 1). A large proportion (38.4%) were classified as having experienced excessive weight gain above the IOM recommended limit for their respective pre-pregnancy body mass index (BMI). Median chronic stress (SSCS-TICS) was 50.0 (25^th^ percentile: 43.0, 75^th^ percentile: 56.0), median HADS-D: 2.0 (1.0, 3.0), and median HADS-A was 4.0 (2.0, 6.0). The respective values for PANX and HCC were median: 15.0 (13.0, 18.0), and 1.74 (1.23, 2.46).

**Table 1:** Characteristics of the study population, N = 748 mother-child pairs.

	Families (N = 748)	
	n/N*	%
**Mothers**		
**Age, years**		
≤25	49/748	6.6
26–35	526/748	70.3
≥36	173/748	23.1
**Nationality** German	632/740	85.4
**Education** ≥12 years	449/732	61.3
**Parity** >1 births	360/748	48.1
**Pre-pregnancy BMI**		
<18.5 kg/m^2^	18/725	2.5
18.5–24.9 kg/m^2^	473/725	65.2
25.0–29.9 kg/m^2^	155/725	21.4
≥30 kg/m^2^	79/725	10.9
Weight gain in kg (cont.) Median, Q1, Q3	13.2, 10.7, 16.3	
**Weight gain according to the IOM categories**		
Inadequate	162/714	22.7
Normal	278/714	38.9
Excessive	274/714	38.4
**Birth mode**		
Vaginal (spontaneous or assisted)	579/748	77.4
Caesarean (elective or emergency)	169/748	22.6
**Children**		
**Sex** male	397/748	53.1
**Birth weight**		
<3000 g	134/748	17.9
3000g–3499 g	330/748	44.1
3500g–3999 g	234/748	31.3
≥4000 g	50/748	6.7

BMI: body mass index, g: gram, IOM: Institute of Medicine, kg/m^2^: kilogram/meter^2^, cont.: continuously measured.

*Number may differ from the overall sum due to missing values.

GEE model results are shown in Table 2. A positive association was observed between chronic stress (SSCS-TICS) reported at childbirth and body weight at first gestational month (regression coefficient (b) = 4.36 kg (95% CI: 1.77; 6.95)) in mothers scoring in the highest tertile (Tert3) of SSCS-TICS compared to those scoring in the lowest tertile (Tert1). Here the first gestational month is thought to reflect pre-pregnancy weight as weight gain in the first three gestational months is generally very low. However, maternal weight gain in women reporting high chronic stress was lower than in their lower stressed counterparts (see Table 2, Test for interaction month*SSCS-TICS tertiles p = 0.043), indicating that the time profiles for those who are highly stressed and those who are not differ. This holds particularly true for months three to nine (see Table 2, column 2 and 4).

**Table 2:** Association of chronic stress (SSCS-TICS tertiles) with gestational weight. Results from a generalized estimating equation model adjusted for covariates (N = 748)^a^.

	Lowest tertile [Tert1] Estimate, 95% CI	Middle [Tert2] Estimate, 95% CI	Highest tertile [Tert3] Estimate, 95% CI
Gestational weight [kg] at first gestational month	Reference	0.76 (−1.39; 2.92)	4.36 (1.77; 6.95)
**Weight gain compared to Month 1**^**b**^			
Month 2	0.31 (−0.11; 0.74)	0.38 (−0.08; 0.84)	−0.19 (−0.78; 0.41)
Month 3	1.59 (1.14; 2.05)	1.62 (1.13; 2.12)	0.94 (0.33; 1.56)
Month 4	3.70 (3.21; 4.19)	3.63 (3.09; 4.16)	2.79 (2.14; 3.43)
Month 5	6.26 (5.72; 6.81)	6.04 (5.47; 6.60)	4.85 (4.18; 5.53)
Month 6	8.49 (7.92; 9.06)	8.19 (7.61; 8.77)	6.87 (6.14; 7.61)
Month 7	10.32 (9.70; 10.93)	10.15 (9.53; 10.77)	8.59 (7.80; 9.38)
Month 8	12.48 (11.82; 13.14)	12.22 (11.53; 12.9)	10.59 (9.73; 11.46)
Month 9	13.95 (13.28; 14.63)	14.14 (13.31; 14.97)	12.51 (11.47; 13.56)

SSCS-TICS: Trier Inventory of Chronic Stress, screening-scale, Tert1: Lowest tertile, Tert2: Middle, Tert3: Highest tertile. ^a^Adjusted for maternal education, maternal age, and maternal height, numbers may differ from the overall N due to missing values.

^b^Interaction (month* SSCS-TICS tertiles).

Depressive symptoms retrospectively reported after delivery were positively associated with maternal body weight at first gestational month (b = 2.50 kg (0.14; 4.86), HADS-D Tert3 vs. HADS-D Tert1, see Table 3) but we did not see an association between depressive symptoms and maternal pregnancy weight gain meaning that women with high depressive symptoms did not gain more or less weight than their non-depressed counterparts. Similarly, there was a statistically significant association between HADS-A and maternal weight at the first gestational month but no significant interaction between maternal anxiety and GWG **(**see Supplementary Table S1**)**. A strong positive relationship between pregnancy-related anxiety and body weight (b = 4.16 kg (1.74; 6.58) PANX Tert3 vs. Tert1, see Table 4) was shown. Furthermore, higher pregnancy anxiety was associated with higher GWG (Test for interaction month*PANX tertiles p = 0.010). Furthermore, a positive association between HCC tertiles and body weight at the first gestational month was observed. Conversely, data revealed an inverse linear relationship (p = 0.147) between HCC and GWG (see Supplementary Table S2**)**. An additional adjustment of the association between SSCS-TICS and gestational weight (gain) by HCC leaded to a small increase of the estimate (regression coefficient (b) = 4.64 kg (95% CI: 2.01; 7.26)) for body weight at first gestational month but no relevant change in the gestational weight gain compared to the model without HCC. Substantial relations between chronic stress or related constructs and IOM GWG category (see Table 5) were only observed for PANX and HCC. High pregnancy-related anxiety was inversely associated with inadequate GWG (adjusted odds OR: 0.59 (95% CI: 0.35, 0.98), Tert3 vs. Tert1), but positively with excessive GWG (OR: 1.70 (95% CI: 1.09, 2.64), Tert3 vs. Tert1). High HCC were significantly associated with higher odds of inadequate GWG (OR: 1.76 (95% CI: 1.06, 2.93), Tert3 vs. Tert1).

**Table 3:** Association of symptoms of depression (HADS-D tertiles) with gestational weight. Results from a generalized estimating equation model adjusted for covariates (N = 748)^a^.

	Lowest tertile [Tert1] Estimate, 95% CI	Middle [Tert2] Estimate, 95% CI	Highest tertile [Tert3] Estimate, 95% CI
Gestational weight [kg] at first gestational month	Reference	−0.88 (−3.12; 1.36)	2.50 (0.14; 4.86)
**Weight gain compared to Month 1**^**b**^			
Month 2	0.24 (−0.19; 0.67)	0.34 (−0.12; 0.81)	−0.04 (−0.59; 0.5)
Month 3	1.42 (0.97; 1.86)	1.60 (1.07; 2.13)	1.25 (0.68; 1.82)
Month 4	3.42 (2.94; 3.91)	3.69 (3.12; 4.26)	3.16 (2.56; 3.76)
Month 5	5.75 (5.22; 6.28)	6.11 (5.47; 6.75)	5.51 (4.90; 6.13)
Month 6	7.89 (7.33; 8.44)	8.33 (7.69; 8.98)	7.60 (6.94; 8.27)
Month 7	9.55 (8.96; 10.14)	10.26 (9.56; 10.96)	9.58 (8.85; 10.30)
Month 8	11.58 (10.95; 12.21)	12.19 (11.43; 12.95)	11.79 (11.00; 12.59)
Month 9	13.32 (12.62; 14.02)	14.39 (13.55; 15.23)	13.39 (12.46; 14.32)

HADS-D: Hospital Anxiety and Depression Scale, Depression, Tert1: Lowest tertile, Tert2: Middle, Tert3: Highest tertile. ^a^Adjusted for maternal education, maternal age, and maternal height, numbers may differ from overall n due to missing values.

^b^Interaction (month* HADS-D tertiles).

**Table 4:** Association of pregnancy related anxiety (PANX tertiles) with gestational weight. Results from a generalized estimating equation model adjusted for covariates (N = 748)^a^.

	Lowest tertile [Tert1] Estimate, 95% CI	Middle [Tert2] Estimate, 95% CI	Highest tertile [Tert3] Estimate, 95% CI
Gestational weight [kg] at first gestational month	Reference	2.08 (−0.26; 4.42)	4.16 (1.74; 6.58)
**Weight gain compared to Month 1**^**b**^			
Month 2	0.15 (−0.34; 0.64)	0.31 (−0.16; 0.78)	−0.02 (−0.54; 0.50)
Month 3	1.44 (0.90; 1.97)	1.47 (0.97; 1.97)	1.29 (0.74; 1.83)
Month 4	3.45 (2.89; 4.00)	3.40 (2.86; 3.94)	3.34 (2.75; 3.93)
Month 5	5.53 (4.92; 6.14)	5.86 (5.27; 6.45)	5.86 (5.25; 6.48)
Month 6	7.64 (7.01; 8.27)	7.93 (7.30; 8.55)	8.07 (7.43; 8.71)
Month 7	9.24 (8.56; 9.92)	9.73 (9.05; 10.42)	10.13 (9.46; 10.81)
Month 8	11.27 (10.54; 12.00)	11.61 (10.86; 12.35)	12.41 (11.66; 13.16)
Month 9	12.92 (12.10; 13.74)	13.38 (12.56; 14.19)	14.36 (13.46; 15.25)

PANX: Pregnancy Related Anxiety Questionnaire, Tert1: Lowest tertile, Tert2: Middle, Tert3: Highest tertile. ^a^Adjusted for maternal education, maternal age, and maternal height, numbers may differ from overall n due to missing values.

^b^Interaction (month*PANX tertiles).

**Table 5:** Association of stress and related constructs with inadequate or excessive gestational weight gain according to the IOM guideline adjusted for potential covariates (N = 748)^a^.

		Inadequate GWG OR (95% CI)	Excessive GWG OR (95% CI)
SSCS-TICS	Lowest tertile [Tert1]	Reference	Reference
	Middle [Tert2]	0.85 (0.52; 1.39)	0.86 (0.56; 1.31)
	Highest tertile [Tert3]	1.23 (0.76; 2.00)	0.85 (0.54; 1.32)
HADS-A	Tert1	Reference	Reference
	Tert2	0.92 (0.56; 1.51)	1.09 (0.70; 1.69)
	Tert3	0.94 (0.56; 1.58)	0.82 (0.52; 1.31)
HADS-D	Tert1	Reference	Reference
	Tert2	0.78 (0.46; 1.32)	0.90 (0.56; 1.44)
	Tert3	0.83 (0.53; 1.31)	1.19 (0.80; 1.79)
PANX	Tert1	Reference	Reference
	Tert2	1.01 (0.63; 1.64)	1.37 (0.86; 2.17)
	Tert3	0.59 (0.35; 0.98)	1.70 (1.09; 2.64)
HCC	Tert1	Reference	Reference
	Tert2	1.46 (0.88; 2.43)	1.11 (0.72; 1.71)
	Tert3	1.76 (1.06; 2.93)	1.35 (0.87; 2.10)

GWG: Gestational weight gain, OR: adjusted odds ratio, SSCS-TICS: Trier Inventory of Chronic Stress, screening-scale, HADS-A: Hospital Anxiety and Depression Scale, Anxiety, HADS-D: Hospital Anxiety and Depression Scale, Depression, PANX: Pregnancy Related Anxiety Questionnaire, HCC: Hair cortisol concentrations, number may differ from overall n due to missing values.

^a^Adjusted for maternal education, maternal age, and maternal pre-pregnancy BMI.

## Discussion

We observed statistically significant positive associations between maternal chronic stress and body weight at the beginning of pregnancy, but a flatter slope of GWG in higher stressed compared to lower stressed mothers. Conversely, higher pregnancy-related anxiety was associated with higher weight at first pregnancy month and higher GWG. Although the directionality of the association between stress, related constructs, and body weight is not entirely clear due to our study design, ‘emotional eating’ acting as a mediator between stress and weight gain during pregnancy could be important and might be considered by practitioners as a target to prevent health consequences of inadequate pregnancy weight gain. We do not have convincing evidence of a mediating role of HCC.

There are several limitations of our study. First, stress and the related constructs were self-reported by the mothers at only one time point just after delivery (median = 1.5 days, range = 0 to 3 days). These measurements may have been subject to recall bias or more proximal delivery-related psychological stress or anxiety. Furthermore, studies of non-pregnant women strongly support the presence of a recursive, bidirectional relationship between nutrition and stress^7,19^. Thus, in spite of our longitudinal study design, we cannot explicitly determine the direction of the association, since stress or related constructs may lead to higher weight gain and vice versa. Further studies should measure stress or related constructs several times throughout pregnancy allowing structural equation modelling and enlighten the complex relationship between stress, related constructs and weight (gain). This idea is also supported by the fact that recently published articles found trimester-specific associations between stress and GWG^20,21^. Additionally, several psychosocial stress instruments differ in their ability to measure prepartal stress^22^. Apart from PANX, our instruments did not account for the specific situation of pregnancy as the instruments were supposed to be used repeatedly in our cohort during long-term follow-up. Thirdly, we did not measure dietary intake and physical activity. Thus, the mechanism behind the relationship between stress and GWG is unknown. Psychological events initiate the HPA axis response resulting in an increased cortisol release, and cortisol, in turn, affects the lipid and carbohydrate metabolism showing a strong association with body weight^23^. Yet, by testing HCC as mediator between SSCS-TICS and weight (gain), our data revealed that HCC were not a significant promotor of weight gain in women. Whether HCC, especially in pregnancy, is therefore a marker of psychosocial stress or, more likely, a marker of metabolic conditions still needs confirmation^24,25^.

A primary strength in our study is the use of objectively measured weight data, documented throughout pregnancy, even though in 57 of our 748 (7.6%) mothers, we used self-reported pre-pregnancy weight as it was also done in many other studies. However, whether the method of measurement can serve as a reason for the inconsistent results reported previously is unclear. Our distribution of GWG was similar to that reported in national observational studies on GWG patterns, with only 30% of women meeting their GWG goals^10^. Alike our results, a strong correlation between pre-pregnancy BMI and excessive GWG^26^ was reported. In our models however, we took pre-pregnancy weight at first gestational month as reference and further adjusted for maternal body height. Pre-pregnancy body mass index is thought to exacerbate unhealthy dietary behavior under high-stress conditions^5^, and stress-induced eating in non-pregnant women with obesity is suggested^27^.

Our data support consistent positive associations between SSCS-TICS, HADS-D, HADS-A, PANX, and HCC and pre-pregnancy body weight, but GWG seems to be lower in mothers scoring high on SSCS-TICS. However, primarily concerning the WHO categories of GWG, one review^20^ reported no convincing association between chronic stress and GWG. Women with high reported stress during pregnancy tended to have low GWG in one study of racially diverse women^28^. Likewise, higher stress was related to inadequate GWG^29^ in African-American women. Conversely, other studies (see review^30^) found no association between chronic stress and low GWG as we did when referring to WHO categories. Given a relatively high socioeconomic status in our study population, we might underestimate the association between chronic stress and inadequate GWG as measured relative to the pre-pregnancy BMI. We did not observe an association for anxiety symptoms and GWG, which is in line with some (see, e.g.,^20^) but not all reports. Hartley *et al*.^12^ showed that higher anxiety was related to greater GWG for 143/256 multiparous women, but, in general, the number of studies on anxiety and weight gain is rather small^31^. Also, covariates including income, health behavior engaged during pregnancy, and familial or social support can modify the association between stress or related constructs and GWG^32^ - factors we could not account for.

Taken together, we conclude that there might be evidence of an association between stress and maternal body weight and weight gain lying beyond what is measured by the IOM categories, which however needs corroboration in other studies. Indeed, further studies should additionally analyse dietary behavior and cortisol to better understand the underling mechanisms between maternal stress, related constructs and pregnancy weight (gain).

## Methods

### Study design and study population

Data were obtained from the Ulm SPATZ Health Study, a birth cohort study recruited from the general population in Ulm, Germany. Mothers who gave birth to a child at Ulm University Medical Center from 04/2012 to 05/2013 and their families were asked to participate (participation rate 49% of eligible mothers, n = 970 with 934 singleton births). Details of the baseline examination are described elsewhere^33^. Exclusion criteria were outpatient delivery, maternal age < 18 years, transfer of the newborn or the mother to intensive care immediately after delivery, insufficient knowledge of the German language, and/or stillbirth. For this analysis, the study population was restricted to singleton full-term (gestational age ≥ 37 weeks) newborns. The ethics board of Ulm University approved the study No. 311/11). All participants gave written informed consent. The study was carried out in accordance with the Declaration of Helsinki.

### Data collection

Maternal demographic and lifestyle data including age at delivery (≤25, 26–35, ≥36 years, nationality (German, other), education (<12 years or ≥12 years), parity (first birth or >1 birth), and maternal occupational status before childbirth (leadership, professional, intermediate position, skilled manual or non-manual, unskilled or semi-skilled, self-employed), were collected using a self-administered questionnaire during the hospital stay following delivery. Clinical data related to the mother’s pregnancy, including gestational body weight measurements and gestational diabetes diagnosis (yes or no), were obtained from paper documentation of routine preventive medical examinations known in Germany as “Mutterpass” (expectant mother’s record of prenatal and natal care), which obstetricians are required to issue to their patients when pregnancy is clinically established and which are generally updated at each clinical visit during pregnancy (preventive medical examinations are suggested in Germany after establishment of pregnancy once a month and after 32^nd^ week of pregnancy every 14 days). Clinical data related to the child’s delivery, including sex and birthweight, were obtained from electronic hospital records, so was the birth mode (vaginal or cesarean).

### Gestational weight gain (GWG)

Pre-pregnancy body weight and height were estimated based on self-reported data recorded by the obstetrician in the Mutterpass typically at the first appointment during which pregnancy was established. GWG was calculated using obstetrician-documented weight measured nearest to the end of each gestational month (n = 906). The start of pregnancy was calculated as 280 days before the obstetrician reported full-term delivery date (“expected birth date”) estimated based on fetal ultrasound measurements at approximately 12 weeks gestation. GWG was additionally categorized into inadequate, adequate, or excessive based on total weight gain (last measured weight before delivery minus first measured weight) and according to the 2009 IOM guidelines^18^ (for body mass index (BMI) < 18.5 kg/m^2^: inadequate: <12.73, adequate: ≥12.73–18.18, excessive: >18.18; for BMI 18.5–24.9 kg/m^2^ <11.36, ≥11.36–15.90, >15.90, for BMI 25.0–29.9 kg/m^2^: <6.82, ≥6.82–11.36, >11.36, and for BMI ≥ 30 kg/m^2^: <5.00, ≥5.00–9.09, >9.09). Data were excluded (n = 57) if the last obstetrician examination had taken place at gestational day 245 or earlier. The weight at the first examination was self-reported body weight for calculation of GWG according to the IOM criteria if the first examination was after the 90^th^ day of pregnancy (n = 52).

### Questionnaire-based stress measurement

Chronic stress during pregnancy was measured using the screening scale of the Trier Inventory of Chronic Stress, (SSCS-TICS)^34^, which assesses chronic concerns, employment-related and social burden, excessive demands, and lack of social recognition experienced during the three prior months. Additionally, the German version of HADS (Hospital Anxiety and Depression Scale), referring to symptoms of anxiety and depression was used^35^. The revised version of the Pregnancy Related Anxiety Questionnaire (PANX)^36^, translated into German, measuring the anxiety of giving birth, the possibility of having a disabled child, and due to self-consciousness related to bodily appearance^37^ experienced throughout pregnancy was additionally used, however, the time span is not explictly specified. For each scale (or subscale) of the aformentioned questionnaires, missing values (TICS: n = 13, HADS D: n = 7, HADS-A: n = 5) were replaced by the mean of the remaining items of the same (sub-) scale.

### Hair cortisol concentrations (HCC)

Hair strands  were taken scalp-near from a posterior vertex position. Cortisol concentrations were determined from the 3 cm hair segment most proximal to the scalp. Based on an average hair growth rate of 1 cm/month^38^, this hair segment represents hair grown over the three months before hair sampling. Wash and steroid extraction procedures of the study followed the laboratory protocol described in detail elsewhere (see^39^).

### Statistical analysis

Firstly, the main characteristics of the study population were described. Then generalized estimating equation (GEE) models were used to incorporate intra-individual correlations of maternal weight throughout time. Thereby, a compound symmetry covariance structure was selected based on the QIC goodness of fit statistics. For predicting gestational weight gain (in kg), the main effect of gestational month and stress or symptoms of mood disorders in tertiles was tested, as well as a stress*gestational month interaction term. Based on reports from the literature^40^, covariates were carefully tested. In the first step, models were adjusted for maternal education, maternal age, maternal height, parity, and smoking during or before pregnancy, maternal occupational status before childbirth, maternal ethnicity, and birthweight. In a second step, parity, maternal occupational status, and ethnicity were excluded from the model, as these variables were not significantly associated (p < 0.1) with the outcome in the adjusted models. So was smoking, a possible marker of maladaptive coping mechanisms. We preferred the model without children’s birthweight, as QIC statistics were smaller after exclusion. In the GEE models, we did not adjust for pre-pregnancy BMI, since weight at the first month was the reference category, which largely corresponds to the pre-pregnancy weight. In an additional analysis, we tested if HCC works as a mediator of the association between SSCS-TICS and weight (gain) according to Baron and Kenny 1986^41^.

For each gestational month, one examination was taken into account. If mothers had more than one examination, the last one in the respective period was chosen. Due to the longitudinal nature of the study, missing data was observed within each gestational month. Of the 906 mothers with gravidogram, no mother had an examination before the end of month 0; 485 (53.1%) had examinations during the first gestational month, 826 mothers (90.5%) in the second, 849 mothers (93.0%) in the third, 847 mothers (92.8%) in the fourth, 847 mothers (92.8%) in the fifth, 873 mothers (95.6%) in the sixth, 896 mothers (98.1%) in the seventh, 860 mothers (94.2%) in the eighth, and 471 mothers (51.6%) in the ninth gestational month. We confirmed that missing weight data were completely at random by conducting bivariate logistic regression analyses specifying missing weight as dependent and the other covariates, including stress and related constructs as independent variables. Thereby, gestational months statistically significant predicted missing values in gestational weight, revealing a lower odds of missing values with higher gestational months.

Furthermore, separate adjusted logistic regression models were used to estimate the association between stress or related constructs and the odds of each low and excessive GWG compared separately to normal GWG with 95% confidence intervals (CI). The logistic regression models were additionally adjusted for initial maternal BMI (categoricaly measured). All statistical analyses were performed with SAS® 9.4 (The SAS Institute, Cary, NC, USA).

## Supplementary information

Supplementary information.

## Data Availability

The datasets generated during and/or analysed during the current study are not publicly available due to ethical restrictions regarding data protection issues and the study specific consent text but are available from the corresponding author on reasonable request.
