# Supplementary material for: Psychosocial stress and longitudinally measured gestational weight gain throughout pregnancy: The Ulm SPATZ Health Study
Source: Sci Rep. 2020 Feb 6;10:1996. doi: 10.1038/s41598-020-58808-8 (PMC7005281; doi:10.1038/s41598-020-58808-8)
Supplement: Supplementary file 1 — Supplementary information. [file 41598_2020_58808_MOESM1_ESM.docx]

**Supplement:**

**Braig S, Logan CA, Reister F, Rothenbacher D, Genuneit J:**

**Psychosocial stress and longitudinally measured gestational weight gain throughout pregnancy: The Ulm SPATZ Health Study**

**Table S1: Association of symptoms of anxiety (HADS-A tertiles) with gestational weight. Results from a generalized estimating equation model adjusted for covariates (N=748)^1^**

|  | **Lowest tertile [Tert1]**  **Estimate, 95% CI** | **Middle [Tert2]**  **Estimate, 95% CI** | **Highest tertile [Tert3]**  **Estimate, 95% CI** |
| --- | --- | --- | --- |
| Gestational weight [kg] at first gestational month | Reference | -1.08 (-3.35; 1.19) | 3.26 (0.62; 5.89) |
| Weight gain compared to Month 1^2^ |  |  |  |
| Month 2 | 0.18 (-0.30; 0.65) | 0.29 (-0.17; 0.76) | 0.03 (-0.50; 0.56) |
| Month 3 | 1.51 (1.00; 2.03) | 1.53 (1.03; 2.02) | 1.20 (0.65; 1.75) |
| Month 4 | 3.51 (2.97; 4.04) | 3.57 (3.03; 4.12) | 3.15 (2.57; 3.74) |
| Month 5 | 5.85 (5.27; 6.43) | 6.03 (5.45; 6.61) | 5.39 (4.78; 6.01) |
| Month 6 | 7.98 (7.35; 8.61) | 8.26 (7.67; 8.85) | 7.42 (6.76; 8.08) |
| Month 7 | 9.60 (8.93; 10.27) | 10.28 (9.64; 10.92) | 9.24 (8.53; 9.95) |
| Month 8 | 11.74 (11.03; 12.45) | 12.29 (11.59; 12.99) | 11.32 (10.53; 12.12) |
| Month 9 | 13.50 (12.73; 14.28) | 14.14 (13.36; 14.93) | 12.95 (11.99; 13.91) |

HADS-A: Hospital Anxiety and Depression Scale, Anxiety, Tert1: lowest tertile, Tert2: middle, Tert3: highest tertile
^1^Adjusted for maternal education, maternal age, and maternal height, numbers may differ from overall n due to missing values

^2^Interaction (month* HADS-A tertiles)

**Table S2: Association of hair cortisol concentrations (HCC tertiles) with gestational weight. Results from a generalized estimating equation model adjusted for potential covariates (N=748)^1^**

|  | **Lowest tertile [Tert1]**  **Estimate, 95% CI** | **Middle [Tert2]**  **Estimate, 95% CI** | **Highest tertile [Tert3]**  **Estimate, 95% CI** |
| --- | --- | --- | --- |
| Gestational weight [kg] at first gestational month | Reference | -0.27 (-2.41; 1.87) | 3.35 (0.86; 5.83) |
| Weight gain compared to Month 1^2^ |  |  |  |
| Month 2 | 0.29 (-0.07; 0.65) | 0.41 (0.11; 0.70) | 0.22 (-0.15; 0.58) |
| Month 3 | 1.64 (1.24; 2.05) | 1.54 (1.21; 1.87) | 1.23 (0.81; 1.65) |
| Month 4 | 3.70 (3.26; 4.14) | 3.46 (3.08; 3.84) | 3.21 (2.73; 3.69) |
| Month 5 | 6.11 (5.61; 6.62) | 5.83 (5.40; 6.26) | 5.41 (4.89; 5.93) |
| Month 6 | 8.16 (7.62; 8.71) | 8.04 (7.57; 8.50) | 7.48 (6.92; 8.05) |
| Month 7 | 10.20 (9.59; 10.82) | 9.83 (9.31; 10.35) | 9.18 (8.56; 9.80) |
| Month 8 | 12.37 (11.69; 13.06) | 11.91 (11.33; 12.50) | 11.10 (10.39; 11.81) |
| Month 9 | 14.15 (13.41; 14.89) | 13.92 (13.26; 14.59) | 13.08 (12.30; 13.87) |

HCC: Hair cortisol concentrations, Tert1: lowest tertile, Tert2: middle, Tert3: highest tertile
^1^Adjusted for maternal education, maternal age, and maternal height, number may differ from overall n due to missing values

^2^Interaction (month* HCC tertiles)
